# Removal of Sulfur Dioxide from Flue Gas Using the Sludge Sodium Humate

**DOI:** 10.1155/2013/573051

**Published:** 2013-12-25

**Authors:** Yu Zhao, Guoxin Hu

**Affiliations:** School of Mechanical and Power Engineering, Shanghai Jiao Tong University, Shanghai 200240, China

## Abstract

This study shows the ability of sodium humate from alkaline treatment sludge on removing sulfur dioxide (SO_2_) in the simulated flue gas. Experiments were conducted to examine the effect of various operating parameters, like the inlet SO_2_ concentration or temperature or O_2_, on the SO_2_ absorption efficiency and desulfurization time in a lab-scale bubbling reactor. The sludge sodium humate in the supernatant after alkaline sludge treatment shows great performance in SO_2_ absorption, and such efficiency can be maintained above 98% with 100 mL of this absorption solution at 298 K (flue gas rate of 0.12 m^3^/h). The highest SO_2_ absorption by 1.63 g SHA-Na is 0.946 mmol in the process, which is translated to 0.037 g SO_2_ g^−1^ SHA-Na. The experimental results indicate that the inlet SO_2_ concentration slightly influences the SO_2_ absorption efficiency and significantly influences the desulfurization time. The pH of the absorption solution should be above 3.5 in this process in order to make an effective desulfurization. The products of this process were characterized by Fourier transform infrared spectroscopy and X-ray diffraction. It can be seen that the desulfurization products mainly contain sludge humic acid sediment, which can be used as fertilizer components.

## 1. Introduction 

Flue gas emissions, which mainly come from power plants by burning fossil fuels, have been causing serious air pollution for decades [1–3]. The reason for serious air pollution caused by flue gas emissions is that flue gas contains large amounts of sulfur dioxide (SO_2_) and other pollutants [4]. Many researchers have been actively exploring technologies in effective flue gas desulfurization field [5–8]. Among these technologies, one of the most effective methods is the wet flue gas desulfurization which is mainly based on limestone [9]. However, it has many disadvantages, such as higher operating costs and greater water requirement and the potential to cause secondary pollution. Thus, cost-effective technologies in removing SO_2_ have become the focus of investigations.

Common commercial sodium humate, which is derived from peat, brown coal, and weathered coal, is a cheap absorbent. Green and Manahan started to use sodium humate to absorb SO_2_ from flue gas in the 1980s [10, 11]. Sun et al. were using humic acid as an additive to modify adsorbents for flue gas desulfurization [12]. Our team used sodium humate solution to remove SO_2_ and NO_*x*_ in flue gas [13, 14]. Sludge sodium humate (SHA-Na) can be extracted from sludge through alkaline treatment method [15]. This paper proposed a new process for the removal of SO_2_ from flue gas by the absorption solution from sludge treatment and the production of fertilizer.

**Figure 1:**
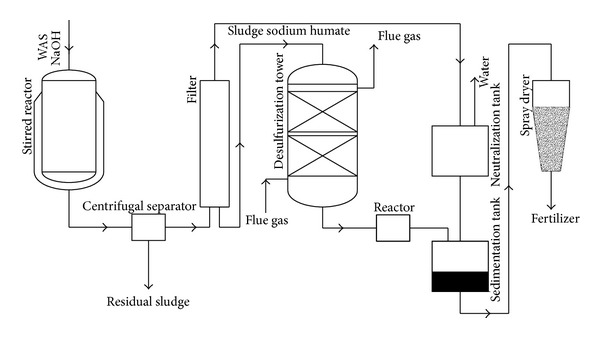
Simplified scheme of application of the SHA-Na for flue gas desulfurization.

The new process shown in Figure 1 includes the following stages. (a) Excess sludge is disintegrated by sodium hydroxide in stirred reactor at 313 K. (b) The disintegrated sludge is centrifuged and the supernatant is concentrated through membrane filter to spray into a desulfurization tower. (c) SO_2_ can be absorbed by SHA-Na in the desulfurization tower. The desulfurization liquid, which mainly contains sludge humic acid (SHA) and H_2_SO_3_, flows into the reactor. (d) In the reactor, SO_3_^2−^ is oxidized to SO_4_^2−^ through diffused aeration. (e) Afterward, the reaction liquid from the reactor flows into a sedimentation tank and stands for 12 h. Due to the poor solubility of SHA, SHA may be separated as sediment from acidic solution. The separated SHA can be used as a kind of material for compound fertilizer after drying in spray dryer. From the above process, it is believed that the removal of SO_2_ by the supernatant from alkaline sludge treatment is a resourceful type of environmental protection technology for FGD. It carries the following advantages: (a) realizes sludge reduction and (b) utilizes the desulfurization product as a useful fertilizer. Therefore, it is hopeful to be applied in the future.

In this paper, we attempt to use a kind of absorption solution from alkaline treatment sludge to remove SO_2 _in flue gas. The effects of the inlet SO_2_ concentration, temperature, and presence or absence of O_2_, on the SO_2_ absorption efficiency, together with desulfurization time in a lab-scale bubbling reactor, are studied. Desulfurization products are characterized by Fourier transform infrared spectroscopy and X-ray diffraction.

## 2. Experimental Section

### 2.1. Materials

Sludge samples were collected from the thickening tank of a wastewater treatment plant. The sludge samples carry water of approximately 98%, a suspended solid (SS) content of 20.0 g/L, a volatile suspended solid (VSS) content of 16.0 g/L, and a soluble chemical oxygen demand (SCOD) of 270 mg/L. Sodium hydroxide (NaOH, AR) was collected from chemical reagent. HA-Na was supplied with purity of 99%. Sulfur dioxide gas with a purity of 99.95% was obtained from the market.

### 2.2. The Absorption Solution Extraction

A slightly modified method [16] was used to extract SHA-Na. The extraction of SHA-Na was conducted in 2.0 L batch mixed reactor that was placed in a water bath to maintain the temperature for this reaction at 313 K while the NaOH is at 0.2 mol/L. Following alkaline sludge treatment, the samples were centrifuged at 5000 r/m for 10 min. After alkaline sludge treatment and centrifugal separation, the sample was filtered through a membrane with a mesh size of 0.45 *μ*m.

### 2.3. Desulfurization Test

A schematic diagram of the experimental setup used in this study is shown in our previous paper [17]. The SO_2_ gas supplied from cylinder was diluted with O_2_ and N_2_ in the gas-mixing chamber. Then, this gaseous mixture flew through the bubbling reactor at ambient pressure. Rota meters and valves were used to monitor the gas flow. The concentrations of the simulated flue gas components (SO_2_ and O_2_) were controlled by adjusting their flow rate and were monitored by a flue gas analyzer. In the progress of this experiment, the total rate of the gas stream was controlled at 0.12 m^3^/h during the experiment.

### 2.4. Characterization Methods

In the experiment, the inlet and outlet concentrations of SO_2_ were measured by a flue gas analyzer. The pH value measuring was conducted with a pH meter using a combination pH electrode PHB-5. Before each pH value was read, the pH buffer solution was used to check the measurements of the electrode. Water content, SS, and volatile dissolved solids (VDS) were measured according to the standard methods [18]. SHA-Na was measured according to the spectrophotometer method recommended by Ye [19]. The protein content was measured using the Coomassie brilliant blue method [20]. FTIR spectra were recorded in the 4000–500 cm^−1^ range using an EQUINOX 55 FTIR spectrometer, continuously purged with dry air. Spectra were obtained from pressed KBr pellets. Pellets (1 cm diameter) were prepared by mixing 1 or 2 mg samples with 200 mg KBr. Desulfurization products were characterized by XRD with a D/max-2200/PC type-ray diffraction instrument to study the composition of them. The heavy metals in products were analyzed by inductively coupled plasma atomic emission spectrometry.

## 3. Results and Discussion

### 3.1. Characteristics of the Supernatant after Alkaline Treatment Sludge

The sludge samples were centrifuged after alkaline treatment, and the supernatant was analyzed. Characteristics of the supernatant after alkaline treatment are shown in Table 1. The supernatant has a high pH value which is similar to the common commercial sodium humate. The VDS indicated that there was a high content of organic substances including sodium humate, protein, polysaccharides, nucleic acid, and lipids [21]. It can be seen that sodium humate and protein were the primary constituents in this supernatant. Sodium humate accounted for approximately 25% of all of the dissolved organic matter by weight. After alkaline sludge treatment, the humic acid concentration in the supernatant was 1.63 g/L. However, the concentration of sodium humate in the absorption solution should be 10~40 g/L in order to get a better absorption solution of SO_2_ in our previous papers. Therefore, when the supernatant was concentrated 10 times by ultrafiltration, the content of sodium humate in the retentate reached the required concentration in the process.

### 3.2. Absorption Process of SO_2_ in the Absorption Solution

**Figure 2:**
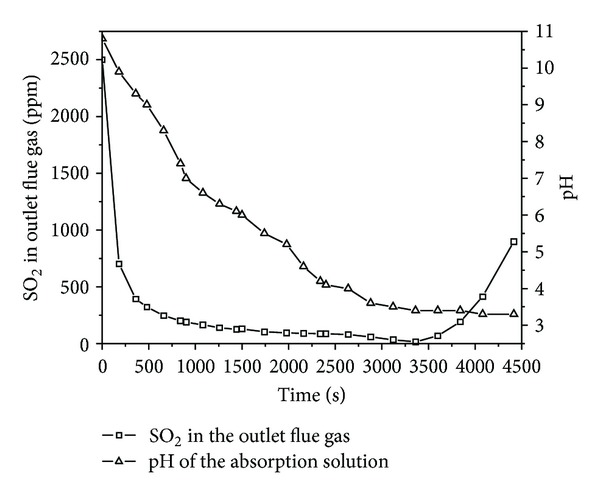
The profile absorption process of SO_2_ in absorption solution. (SO_2_, 2500 ppm; gas flow, 0.12 m^3^/h; absorption solution 100 mL; O_2_, 5 vol%; 298 K).

The change of the SO_2_ concentration in outlet flue gas with time is shown in Figure 2. It can be observed that the SO_2_ absorption curve in absorption solution is divided into three sections: a descending section, a nearly horizontal section, and an ascending section. Also, the pH of the absorption solution in this process was clearly illustrated in Figure 2. The change of pH in the absorption solution is related to the SO_2_ concentration in outlet flue gas. In the descending section of SO_2_ absorption curve, the pH value of the absorption solution drops rapidly from 10.8 to 7.4, because of the faster consumption of OH^−^ in the absorption solution. The pH value of the absorption solution decreases slowly from 7.4 to 3.4 in the nearly horizontal section of SO_2_ absorption curve, as the SHA-Na in the absorption solution is a sort of pH buffer solution [22], this behavior was interpreted as a buffer action of –COOH [23], which may lower the rate of pH value decrease. In the ascending section of SO_2_ absorption curve the pH value of SHA-Na maintains a constant number. It can be explained as follows: the absorption solution loses the desulfurization capability as the SHA-Na in the absorption solution has converted to SHA sediment. From the above analyses, it can be seen that when the pH value of the absorption solution drops to 3.4, the absorption solution will loose the desulfurization capability. Hence, the pH value of the absorption solution should be above 3.4 in this process in order to make it an effective desulfurization.

### 3.3. Analysis of the Desulfurization Mechanism

In removing SO_2_ process with this absorption solution, the main reactions are that SO_2_ from simulated flue gas react with SHA-Na, which is an acid-base reaction, and the acid-base theory predicts that SHA-Na should react with SO_2_ by following neutralization reaction [10, 11]:

(1) SHA-Na(aq)+SO2(g)+H2O ⟷SHA(s)+HSO3−(aq)+Na+(aq)

Simultaneously, when SO_2_ is dissolved in the absorption solution, based on its alkalinity, the following reactions occur [24, 25]:

(2) $$\begin{array}{c} {\text{SO}}_{2}\left(\text{g}\right)⟷{\text{SO}}_{2}\left(\text{aq}\right) \end{array}$$

(3) $$\begin{array}{c} {\text{SO}}_{2}\left(\text{aq}\right)+{\text{H}}_{2}\text{O}⟷{\text{H}}_{2}{\text{SO}}_{3}\left(\text{aq}\right) \end{array}$$

(4) $$\begin{array}{c} {\text{H}}_{2}{\text{SO}}_{3}\left(\text{aq}\right)⟷{\text{H}}^{+}\left(\text{aq}\right)+{{\text{HSO}}_{3}}^{-}\left(\text{aq}\right) \end{array}$$

(5) $$\begin{array}{c} {{\text{HSO}}_{3}}^{-}\left(\text{aq}\right)⟷{\text{H}}^{+}\left(\text{aq}\right)+{{\text{SO}}_{3}}^{2-}\left(\text{aq}\right) \end{array}$$

Generally, the above reactions can be described by the following equilibrium equations (6) and (7):

(6) $$\begin{array}{c} {\text{SO}}_{2}\left(\text{aq}\right)+{\text{OH}}^{-}⟷{{\text{HSO}}_{3}}^{-}\left(\text{aq}\right) \end{array}$$

(7) $$\begin{array}{c} {{\text{HSO}}_{3}}^{-}\left(\text{aq}\right)+{\text{OH}}^{-}⟷{{\text{SO}}_{3}}^{2-}\left(\text{aq}\right)+{\text{H}}_{2}\text{O} \end{array}$$

Then, SO_3_^2−^ is oxidized to SO_4_^2−^ by catalysis with transition metal ions, which is the step for (6) and (7) to move to the right. The dissolved SO_2_ also produced H^+^ by ionization in this process. The acidic groups of SHA-Na, such as carboxyl (COO^−^) and hydroxyl (OH^−^), can react rapidly with H^+^, and SHA-Na is transferred to sludge humic acid. According to (1) and (5), this reaction also moves the reaction equilibrium to the right, which results in the fact that more SO_2_ is dissolved into the absorption solution. When entire SHA-Na is consumed, the desulfurization reaction is terminated in the process.

### 3.4. Desulfurization Performance of SHA-Na

Several additional experiments were done to gain a further understanding of desulfurization performance of SHA-Na. The contrast experiments of removing SO_2_, one used water at the same volume and another used NaOH solution at the same pH (10.8), were conducted, respectively. Total SO_2_ absorption can be calculated by (8)

(8) $$\begin{array}{c} Q=\overset{T}{\underset{0}{\int }}\frac{\left(\eta \times q\times C_{0}\times M\right)}{22.4\times 3600\times 10^{3}}dt,\\ Q=\frac{q\times C_{0}\times M}{8.064\times 10^{7}}\overset{T}{\underset{0}{\int }}\eta \;dt, \end{array}$$

where *Q* stands for the amount of SO_2_ absorption, mmol and *η* stands for the removal efficiency of SO_2_, %. The formula of the SO_2_ absorption efficiency can be obtained from the literature [17]. *q* stands for the gas flow, m^3^/h; *C*_0_ stands for the inlet concentration of SO_2_, ppm; *M* stands for the molar mass SO_2_, g/mol; *T* stands for the reaction time, s.

It is clear as shown in Table 2 that experimental results illustrate that SHA-Na performance much better than that of water and NaOH in both the SO_2_ absorption efficiency and the duration time of high efficiency ((DTHE), the time of the SO_2_ absorption efficiency is above 70%). Water absorbs the SO_2_ more slowly because it is only a physical absorption process controlled by molecular diffusion [26]. However, when we use SHA-Na in this absorption solution to remove SO_2_, the hydroxyl radicals in SHA-Na can react rapidly with the SO_2_. Thus, it leads to the decrease of the SO_2_ concentration in the gas-liquid interface. The SO_2_ diffusion can be promoted due to this decrease of the SO_2_ concentration in the gas-liquid interface. Furthermore, SHA-Na can react with H^+^ as what common commercial sodium humate does, and it is transformed into humic acid sediment. Therefore, it can be concluded that the desulfurization capability of SHA-Na mainly depends upon the consumption of the hydroxyl radicals as well as common commercial sodium humate. Compared with NaOH solution, it is also shown in Table 2 that the desulfurization capability of SHA-Na is stronger than NaOH solution at the same volume and pH value in Table 2. It can be explained that the hydroxyl radicals of NaOH solution are less than those of SHA-Na at the same volume and pH value. The contrast experiments of removing SO_2_ with different sludge as the absorption solution at the same volume are also conducted, respectively. The experimental results illustrate that SHA-Na performance is also much better than that of activated sludge and excess sludge in both the SO_2_ absorption efficiency and DTHE. It can be explained that the SO_2_ absorption efficiency and desulfurization time depend on the content of fulvic acid that can excellently absorb SO_2_ in the activated sludge or excess sludge [25]. The content of fulvic acid in the activated sludge is lower than the content of fuluic acid in the excess sludge, so the SO_2_ absorption efficiency of the activated sludge is lower than that of the excess sludge and the desulfurization time of the activated sludge is shorter than that of the excess sludge. Therefore, SHA-Na can be practically used as a kind of absorbent for the removal of SO_2_.

### 3.5. Effect of the Inlet SO_2_ Concentration

**Figure 3:**
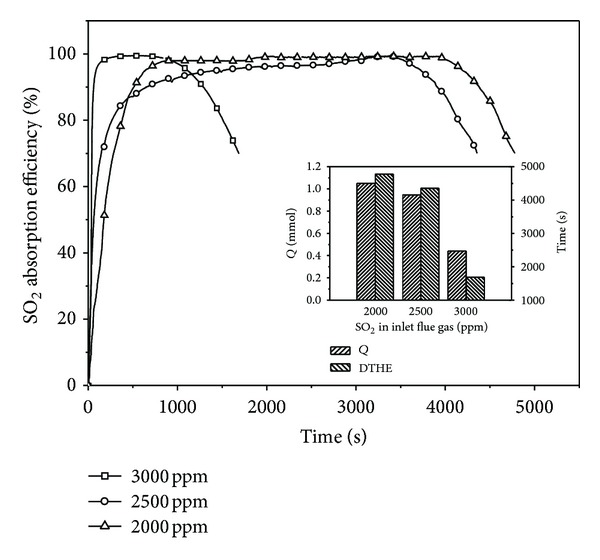
The effect of the inlet SO_2_ concentration (gas flow, 0.12 m^3^/h; absorption solution, 100 mL; O_2_, 5 vol%; 298 K). The inset shows the absorption amount of SO_2_ and DTHE.

The effect of the inlet SO_2_ concentration on absorption efficiency is displayed in Figure 3. The inlet SO_2_ concentration does not have significant effect on the SO_2_ absorption efficiency under this condition, and the SO_2_ absorption efficiency showed a constant value of about 98% in nearly horizontal section. However, the desulfurization time in this section decreases greatly with the increase of the inlet SO_2_ concentration. This can be explained as follows: according to the model [27], the absorption of SO_2_ requires the removal of aqueous SO_2_ from the gas-liquid interface. The reaction with SHA-Na produced by sludge allows the concentration of SO_2_ in the absorption solution to decrease. However, the absorption effect of SO_2_ becomes worse because of higher concentration of SO_2_ at the gas-liquid interface when the inlet SO_2_ concentration increases. Hence the desulfurization time in the nearly horizontal section decreased. And the absorption amount of SO_2_ at the inset in Figure 3, which decreases with the increase of the inlet SO_2_ concentration, shows a reasonable agreement with it.

### 3.6. Effect of the Temperature

**Figure 4:**
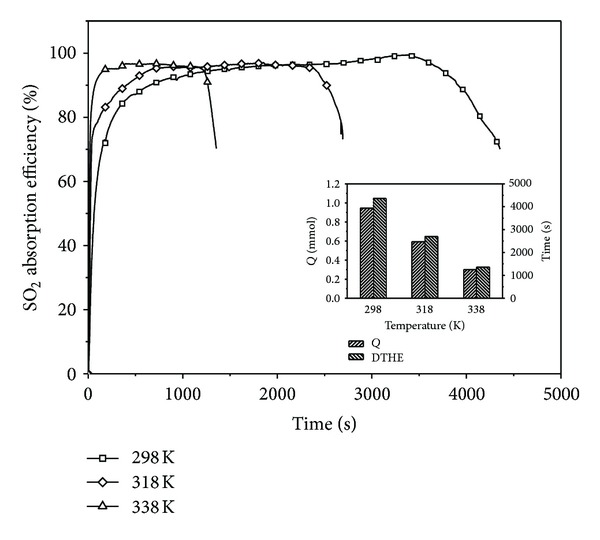
The effect of the temperature (SO_2_, 2500 ppm; gas flow, 0.12 m^3^/h; absorption solution, 100 mL; O_2_, 5 vol%). The inset shows the absorption amount of SO_2_ and DTHE.

The effect of the temperature on the SO_2_ absorption efficiency was also conducted. The experimental results are shown in Figure 4. It indicates that the absorption temperature has slight effect on the SO_2_ absorption efficiency and the desulfurization time in the nearly horizontal section becomes shorter with the rise of the temperature. The SO_2_ absorption efficiency increases slightly as the absorption temperature ascends from 298 K to 338 K. However, the desulfurization time in the nearly horizontal section decreases from 3500 s to 1300 s as the absorption temperature ascends from 298 K to 338 K. It can be explained as follows. On one hand, according to the dissolution equilibrium of SO_2_, the equilibrium partial pressure of SO_2_ is increasing with the rise of the temperature, which results in the escape of some dissolved SO_2_ from solution. In addition, the limit of “diffusional regime” is probably reached and this limit depends on temperature proposed by Lancia and Musmarra [28]. The solubility of SO_2_ decreases with the rise of the absorption temperature in water. On the other hand, the contact time of flue gas and liquid is reduced because the relative velocity of gas molecules speeds up at high temperature. Hence, the absorption amount of SO_2_ decreases with the increase of the temperature at the inset in Figure 4. Although the removal of SO_2_ is promoted at low temperature, removing SO_2_ with SHA-Na solution in the ambient condition at room temperature should be suitable for this process to reduce the desulfurization cost.

### 3.7. Effect of Oxygen

**Figure 5:**
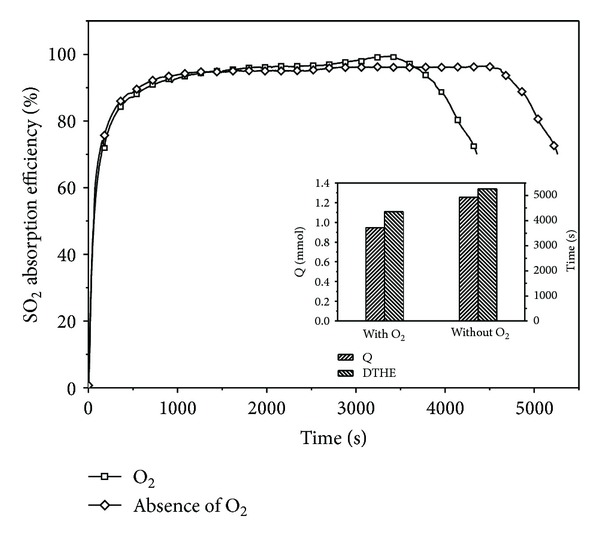
The effects of O_2_ (SO_2_, 2500 ppm; gas flow, 0.12 m^3^/h; absorption solution, 100 mL; 298 K). The inset shows the absorption amount of SO_2_ and DTHE.

Flue gas from power plant usually consists of about 5 vol% O_2_, trace gases, 15 to 20 vol% CO_2_, and balance N_2_ [29]. Hence, oxygen (a concentration of 5 vol%) was introduced into a simple simulated flue gas (N_2_ + SO_2_), that is, the simulated flue gas for this study, to understand the potential influence of the presence of O_2_ on the SHA-Na performances and interaction of SO_2_ and SHA-Na in the absorption solution. Test was conducted without the presence of O_2_, where relative to the presence of O_2_, which is shown in Figure 5. The SO_2_ absorption efficiency has a slight decrease under an aerobic condition, while the absorption amount of SO_2_ and DTHE has little increase. It is known that the dissolved O_2_ in the absorption solution may accelerate the oxidation of sulfite into sulfate. Sulphite oxidation rate can be greatly improved by very low metal concentration [30, 31]. The mass-transfer resistance of the liquid phase in absorption solution is lessened, which results in the promotion of the SO_2_ mass transfer. Thus, the SO_2_ absorption efficiency with the presence of oxygen is higher than that with the absence of O_2_. However, it maybe that the consumption of O_2_ makes a corresponding growth of the inlet SO_2_ concentration, which results in the lower absorption amount of SO_2_ and DTHE. Based on the flue gas, the oxygen concentration was maintained at 5% in the simulated flue gas of N_2_, O_2_ and for subsequent experiments.

### 3.8. Analysis of Desulfurization Product

**Figure 6:**
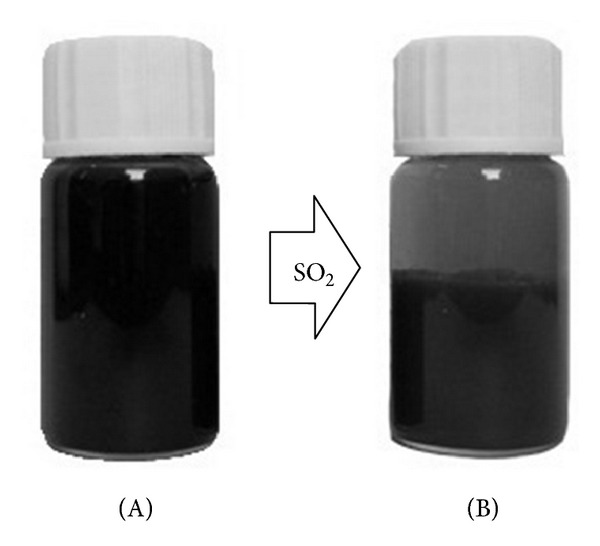
The photo of (A) SHA-Na solution; (B) desulfurization liquid after standing for 3 hours (SO_2_, 2500 ppm; gas flow, 0.12 m^3^/h; absorption solution, 100 mL; O_2_, 5 vol%; 25°C).

After the desulfurization process, the absorption liquid shown in Figure 6 was separated into liquid and solid by filtration. The crystals were gained by drying the supernatant layer from the absorption liquid.

**Figure 7:**
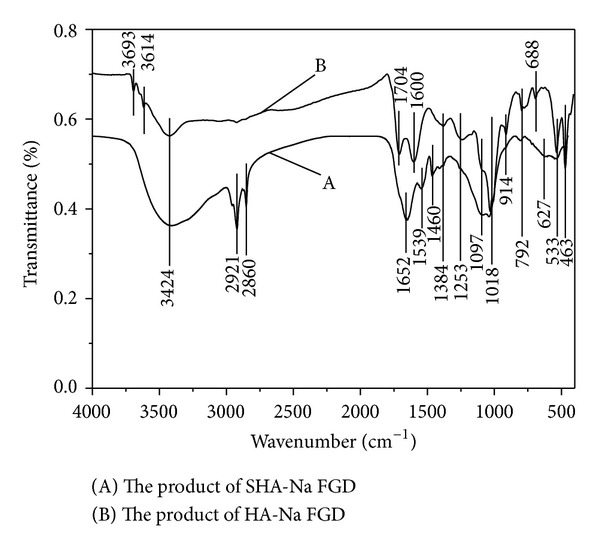
FTIR spectra of desulfurization product (SO_2_, 2500 ppm; gas flow, 0.12 m^3^/h; absorption solution, 100 mL; O_2_, 5 vol%; 25°C).

**Figure 8:**
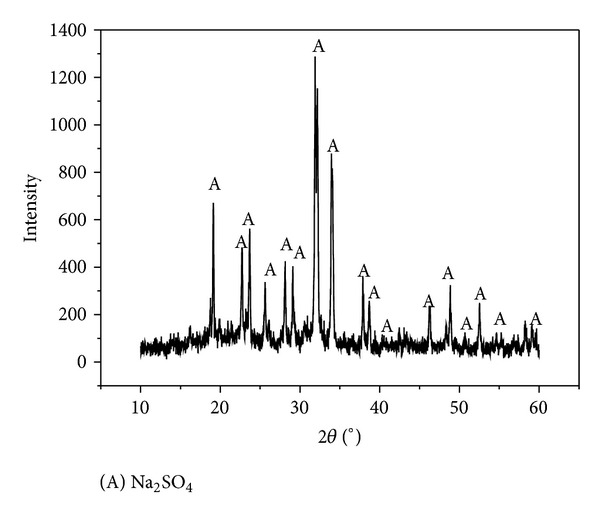
XRD pattern of the crystals from supernatant (SO_2_, 2500 ppm; gas flow, 0.12 m^3^/h; absorption solution, 100 mL; O_2_, 5 vol%; 25°C).

FTIR spectroscopy is a powerful and nondestructive tool for the investigation of decomposition in the desulfurization product.  Figure 7 shows the FTIR spectra comparison diagram of desulfurization products which are from SHA-Na desulfurization process and HA-Na desulfurization process, respectively. The main absorbance bands of them are in common a broad band at the wavenumbers of 3424 cm^−1^ (H bonds, OH groups), 1384 cm^−1^ (COO^−^, CH_3_), a small peak at 1253 cm^−1^ (aromatic C, C–O stretch), a slight shoulder around 1097 cm^−1^ (aliphatic CH_2_, OH, or C–O stretch of various groups), and a peak at 1050 cm^−1^ (C–O stretch of polysaccharide) [32, 33]. Compared with spectra of humic acid, it is obvious that distinct differences in the spectra that resulted from the SHA-Na flue gas desulfurization process appear as peaks in the aliphatic region at 2921 and 2860 cm^−1^, [34] in which sludge humic acid contains a lot of fat material. Three new bands (1652, 1539, and 1460 cm^−1^) appear in the 1700–1450 cm^−1^ spectra of all the products of desulfurization. HAs are distinguished by the presence of a strong absorption band near 1650 cm^−1^, moderately strong absorption at 1540 cm^−1^, strong absorption near 1050 cm^−1^, and relatively pronounced absorption near 2900 cm^−1^ [35]. A unique feature of this spectrum also shows the presence of bands indicated proteins and carbohydrates. Due to its poor solubility, sludge humic acid may be separated as sediment from acidic aqueous solution and converted into humic acid compound fertilizer. The chief product of drying the supernatant layer was characterized by XRD. As shown in Figure 8, the diffraction peaks at the XRD patterns of the catalysts obviously belong to Na_2_SO_4_ [36, 37].

Heavy metals are a limiting factor for sludge fertilizer. The heavy metal content of humic acids extracted from sludge depends on the dissolution rate of sludge heavy metals and the rejection rate of heavy metals by the membrane. During NaOH treatment, the dissolved heavy metals in sludge generally comprise less than 60% of the total heavy metals [38]. Under the strong alkaline conditions, only part of the amphoteric metal in proper forms can be dissolved. Therefore, the heavy metal content in the supernatant was low in Table 3. It can be seen that after the desulfurization process, the desulfurization product contains mainly sludge humic acid sediment, which may be used as fertilizer components.

## 4. Conclusion

In the present work, alkaline pretreatment of the activated sludge represented a suitable and effective method for the preparation of SHA-Na. The characteristics of SO_2_ absorption into the SHA-Na absorption solution have been investigated in a bubbling reactor. The experimental results show, that compared with water (with the same volume) and NaOH solution (with the same pH), SHA-Na shows greater performance in SO_2_ absorption. The effects on the SO_2_ absorption efficiency and desulfurization time have been studied, while the inlet SO_2_ concentration and temperature were changed. Characterization of the desulfurization products was performed by FTIR and XRD analysis. It was found that the desulfurization products can be used as the compound fertilizer. The concept of combat environmental problems by methods such as removing a waste by using another waste source is of economic interest. Therefore, it is possible for the desulfurization process of SHA-Na to be used in future.

## Figures and Tables

**Table 1:** Characteristics of the supernatant after alkaline treatment.

Parameters	Value	Organic substances	Value	Proportion (%)
pH	10.8	Sodium humate	1.63 g/L	24.1%
SS	0.07 g/L	Protein	2.0 g/L	29.6%
VDS	6.756 g/L	Othersa	3.126 g/L	46.3%

**Table 2:** The SO_2_ absorption using different absorption solution^a^.

Sample	Maximum absorption efficiency of SO_2_ (%)	DTHE (s)	*Q* (mmol)
SHA-Na	98.32	4357	0.946
NaOH	81.04	478	0.080
H_2_O	78.52	240	0.038
Activated sludge	90.32	408	0.075
Excess sludge	92.40	840	0.166

^a^ *C*_0_: 2500 ppm; *q*: 0.12 m^3^/h; absorption solution: 100 mL; O_2_: 5 vol%; 298 K; ambient pressure.

**Table 3:** The contents of humic acid and heavy metals in the product and the comparison with the standard.

Indexes	In the standard	In the product
Humic acid (g/L)	≥30	15.2
As (mg/L)	≤10	0.56
Cd (mg/L)	≤10	0.41
Pb (mg/L)	≤50	5.31

(SO_2_: 2500 ppm; gas flow: 0.12 m^3^/h; absorption solution: 100 mL; O_2_: 5 vol%; 25°C.)
